# *In situ* printing of mesenchymal stromal cells, by laser-assisted bioprinting, for *in vivo* bone regeneration applications

**DOI:** 10.1038/s41598-017-01914-x

**Published:** 2017-05-11

**Authors:** Virginie Keriquel, Hugo Oliveira, Murielle Rémy, Sophia Ziane, Samantha Delmond, Benoit Rousseau, Sylvie Rey, Sylvain Catros, Joelle Amédée, Fabien Guillemot, Jean-Christophe Fricain

**Affiliations:** 1 University of Bordeaux, Tissue Bioengineering, U1026, F-33076 Bordeaux, France; 2 Inserm, Tissue Bioengineering, U1026, F-33076 Bordeaux, France; 3 ART BioPrint, Inserm, U1026, F-33076 Bordeaux, France; 4 CHU Bordeaux, Services d’Odontologie et de Santé Buccale, F-33076 Bordeaux, France; 5 Clinical Research Center - Technological Innovation, Inserm, Bordeaux University Hospital, Pessac, 33600 France; 6 Animalerie A2, Université Bordeaux Segalen, Bordeaux, France

## Abstract

Bioprinting has emerged as a novel technological approach with the potential to address unsolved questions in the field of tissue engineering. We have recently shown that Laser Assisted Bioprinting (LAB), due to its unprecedented cell printing resolution and precision, is an attractive tool for the *in situ* printing of a bone substitute. Here, we show that LAB can be used for the *in situ* printing of mesenchymal stromal cells, associated with collagen and nano-hydroxyapatite, in order to favor bone regeneration, in a calvaria defect model in mice. Also, by testing different cell printing geometries, we show that different cellular arrangements impact on bone tissue regeneration. This work opens new avenues on the development of novel strategies, using *in situ* bioprinting, for the building of tissues, from the ground up.

## Introduction

Much attention has been drawn to tissue engineering approaches in order to build three-dimensional (3D) constructs to replace or sustain the regeneration of tissues, using a combination of biocompatible and bioactive biomaterials with/or without cells and bioactive factors^1^. Two major tissue-engineering manufacturing approaches have been implemented: the top-down and the bottom-up approaches^2^. In top-down approaches, cells are often seeded sparsely within a synthetic or natural scaffold (or a decellularized tissue) shaped to adapt to the desired geometry of the lesion to regenerate. This construct is often matured in a bioreactor. Over the past two decades, this approach has led to some scientific progress and preclinical success, especially for the regeneration of thin or avascular tissues, such as skin^3^, cartilage or connective tissues^4^, or tissues with a high capacity to regenerate or remodel, such as bone^5^. However, top down approaches hardly mimic the intricate microstructural features of native tissues, and the colonization and differentiation of cells within these scaffolds remains difficult to control. Indeed, regarding clinical applications, only few clinical trials have been reported and shown to succeed^6^.

On the other hand, bottom up approaches, based on a brick-by-brick reconstruction of a tissue, offer the opportunity to pattern the individual components according to a predefined pattern that will guide the maturation of the tissue construct towards a final functional architecture. As a result, cellular distribution can be defined at the micrometer scale, enabling the creation of a proper extracellular matrix (ECM) microenvironment. Among different bottom-up techniques, bioprinting, an emerging advanced biofabrication method, has since the last five years become one of the most promising technical approach in order to attain control over the geometry of engineered tissues. This approach takes advantage of rapid prototyping, assisted by computer-assisted design (CAD) and/or computer-assisted manufacturing (CAM) procedures, to build a cellularized scaffold with geometric control of its internal structure and external shape^7^.

To date, three major bioprintng techniques have been established: inkjet, laser assisted and extrusion. Numerous reviews have reported a concise comparison of these three strategies^7–10^. Among these, Laser-Assisted Bioprinting (LAB) technology has emerged, from the initial work and patents of researchers at the Naval Research Laboratory^11^, as a promising method for engineering artificial tissues. This latter method is based on the laser-induced forward-transfer (LIFT) effect. LIFT Assisted Bioprinters or LAB are composed of 3 main constituents: (1) a pulsed laser source, (2) a target, or ribbon, serving as a support for the printing material, and (3) a support to collect the printed material. Briefly, the ribbon is composed by a support, that is non absorbing to the laser (*e.g*. glass or quartz), coated by a thin laser absorbing layer of metal (*e.g*. gold or titanium). The organic components (cells or molecules) are prepared inside a liquid phase (*e.g*. culture medium or collagen), and deposited on the surface of the metal-coated support. Then, the laser pulse induces the vaporization of the metal film, resulting in the formation of a droplet, which is then deposited on the receiving substrate^12, 13^. LAB is a direct-write method that can manage droplet deposition of cells or biomaterials, within a fluidic phase, at an MHz range speed. Due to its picoliter-level resolution, LAB allows to control the cell density and spatial 3D organization, up to the single cell level, enabling unprecedented control over cell behavior and fate, key parameters in tissue engineering. As such, LAB is an emerging and promising technology to fabricate tissue-like structures with the capacity to mimic the physiological functionality of their native counterparts. Additionally, this method has additional advantages such as automation, reproducibility, and high throughput, making it compatible with the fabrication of 3D constructs of physiologically relevant sizes.

Previously we focused on this bioprinting approach for *in vivo* computer-assisted medical interventions and on an *in situ* tissue engineering application^14^. For that purpose, a workstation dedicated to high-throughput biological laser printing was adapted to *in vivo* printing experiments. The proof of concept for *in vivo* printing was already performed using LAB by a deposition of particles of hydroxyapatite (HA) into a mouse calvaria defect of critical size, *in vivo* ^14^. In this work, and using LAB technology, we focus on the impact of different geometric cell patterning in order to achieve guided regeneration of *in vivo* bone tissue, following bioprinting. We show, for the first time, the use of LAB technology for the regeneration, *in situ*, in a critical size bone defect, by printing different biological components and mesenchymal stromal cells in a well-defined pattern.

## Laser assisted Bioprinting (LAB)

**Figure 1:**
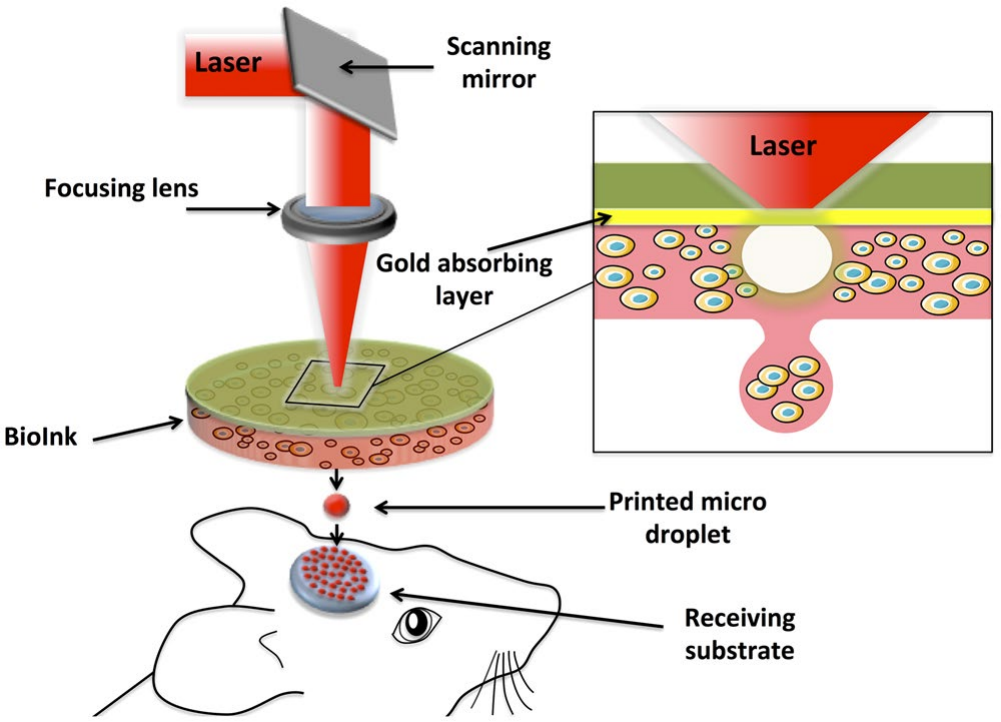
Schematic representation of the laser assisted bioprinting (LAB) approach. A typical LAB setup comprises a pulsed laser beam, a focusing system, a ribbon (a transparent glass slide, coated with a laser-absorbing layer of metal, onto which a thin layer of bioink is spread, and a receiving substrate facing the ribbon. The physical principle of LAB is based on the generation of a cavitation-like bubble, into the depth of the bioink film, whose expansion and collapse induces the formation of a jet and, thereby, the transfer of the bioink from the ribbon to the substrate (here a bone defect on the mouse calvaria), forming a microdroplet.

The bioprinting approach used in this work was based on LAB, using a workstation dedicated to high-throughput biological laser printing adapted to *in vivo* use. The applied setup, previously described^15^, is based on a near infra red pulsed laser beam coupled to a scanning mirror and a focusing system (Fig. 1). This setup allows to precisely focus a laser bean on the ribbon (a transparent quartz glass slide coated with a gold absorbing layer), onto which a thin layer of cellularized ink is spread. The energy created by the incidence of the laser beam creates a cavitation that propels a microdroplet, containing cells, towards the receiving substrate, that can be a 2D support or an exposed 3D *in vivo* tissue (Fig. 1).

**Figure 2:**
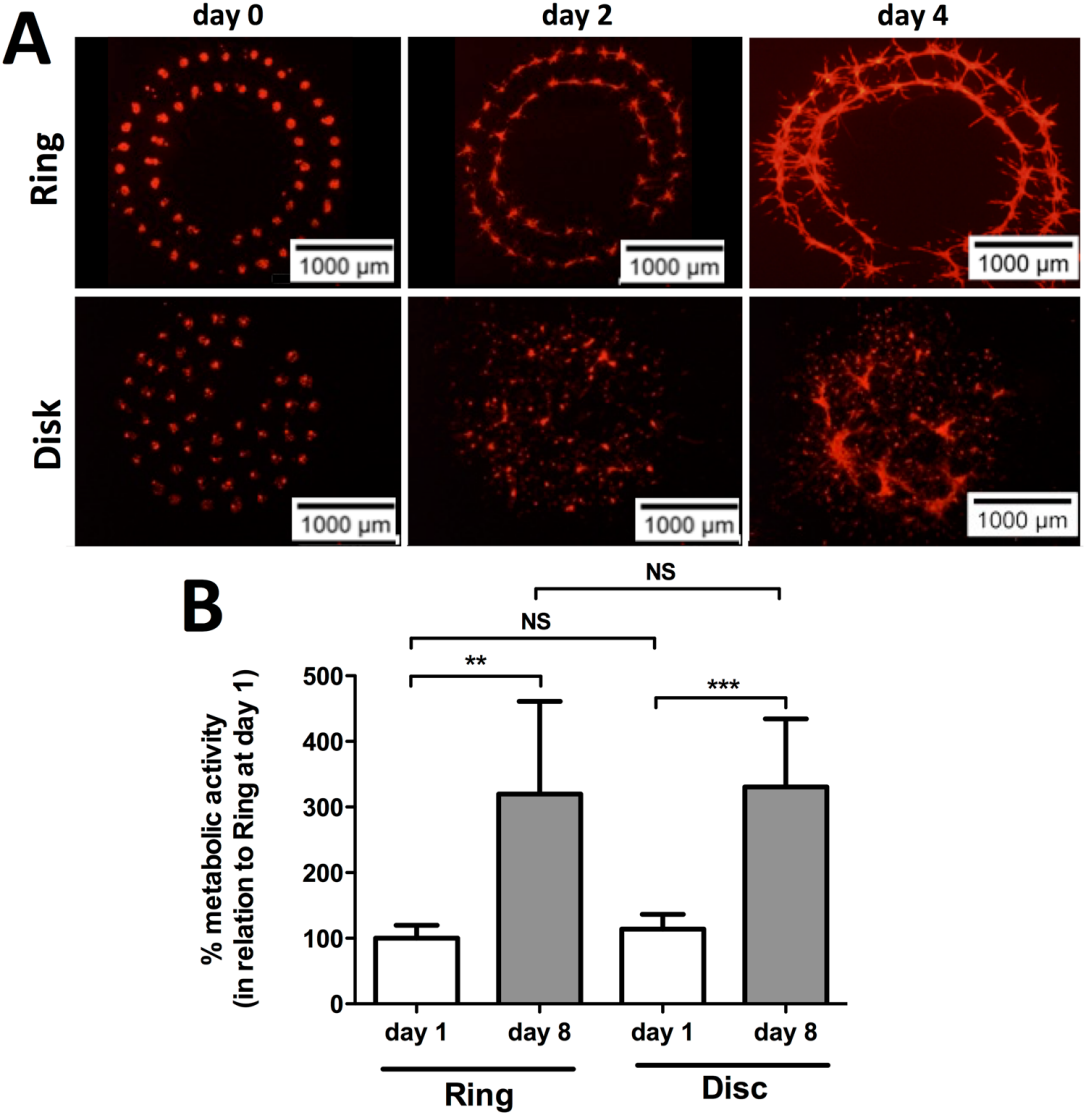
(**A**) Representative fluorescence images of ring and disk printed tomato-positive D1 cells at days 0, 2 and 4. (**B**) Percent metabolic activity, as measured by the resazurin assay, of D1 cells printed in a ring or disk geometry at days 1 and 8, in relation to ring geometry at day 1 (Average ± SD, n = 6, **and ***denotes p < 0.01 and p < 0.001, respectively).

In view to establish the conditions to be used in the subsequent *in vivo* studies, we evaluated the *in vitro* cellular response of D1 cells (multipotent mouse bone marrow stromal precursor cells) to two different printed designs: a ring and a disk. Our rational aimed to further understand the impact of cellular distribution *in vivo*, upon bioprinting by LAB, on the regeneration of a bone defect in mice. As such, tomato positive D1 cells were printed, using two distinct geometries but with the same total number of cells, over a collagen substrate and their morphology was followed up to 4 days. As seen in Fig. 2A, both geometries show an adequate correspondence to the predefined design after printing. Also, with time of culture, cells show to spread/proliferate and fill the voids between the spots, at days 2 and 4 (Fig. 2A).

As means to establish the behavior of D1 cells upon printing we evaluated the metabolic activity at both 1 and 8 days, relative to the ring geometry at day 1, post-printing (Fig. 2B). Cells show a significant increase in terms of metabolic activity from day 1 to 8, for both geometries (Fig. 2B). Also, no significant differences could be observed between the two different geometries, at the same time point, asserting for the consistence of the printing (Fig. 2B).

## ***In vivo*** and ***in situ*** Bioprinting

**Figure 3:**
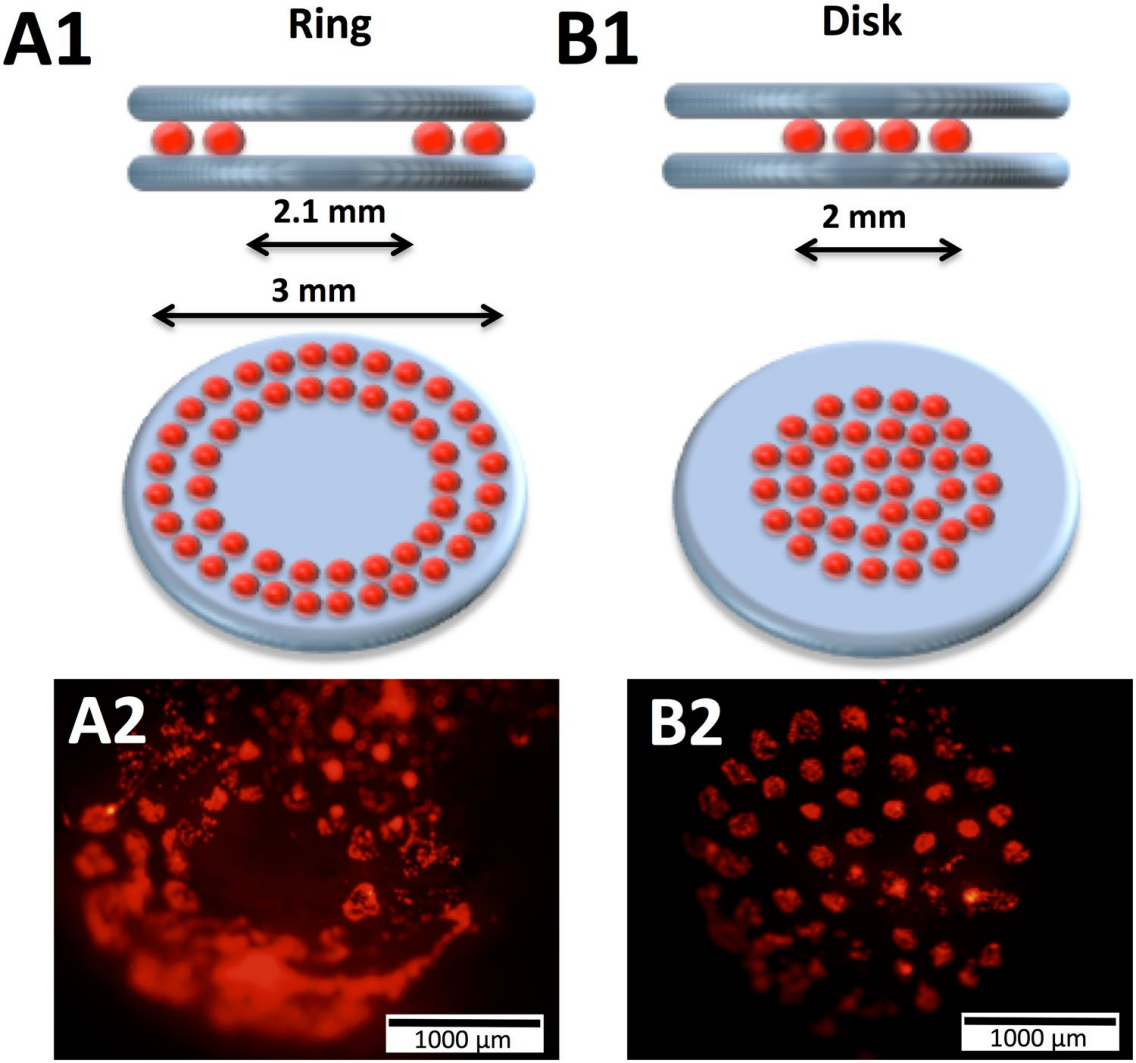
Schematic representation of the *in vivo* laser assisted bioprinting geometries tested, namely a ring (**A1**) with external and internal diameter of 3 and 2.1 mm, respectively, and a disk (**B1**) with 2 mm diameter. In both cases, two layers of nHA-collagen ink were printed underneath and over the cellularized ink layer. Representative fluorescence images of ring (**A2**) and disk (**B2**) printed tomato-positive (**D1**) cells inside the calvaria defect in mice, immediately after printing.

Based on the printing designs tested in *in vitro* conditions, we then followed to adapt the same printing geometries to an *in vivo* defect in mouse calvaria. In order to confine the printed cell spots to the calvaria defect, and to provide an osteoconductive matrix to the printed cells, two nHA-collagen disks were printed before and after the cellularized ink printing (Fig. 3A1 and B1, for ring and disk geometries, respectively).

**Figure 4:**
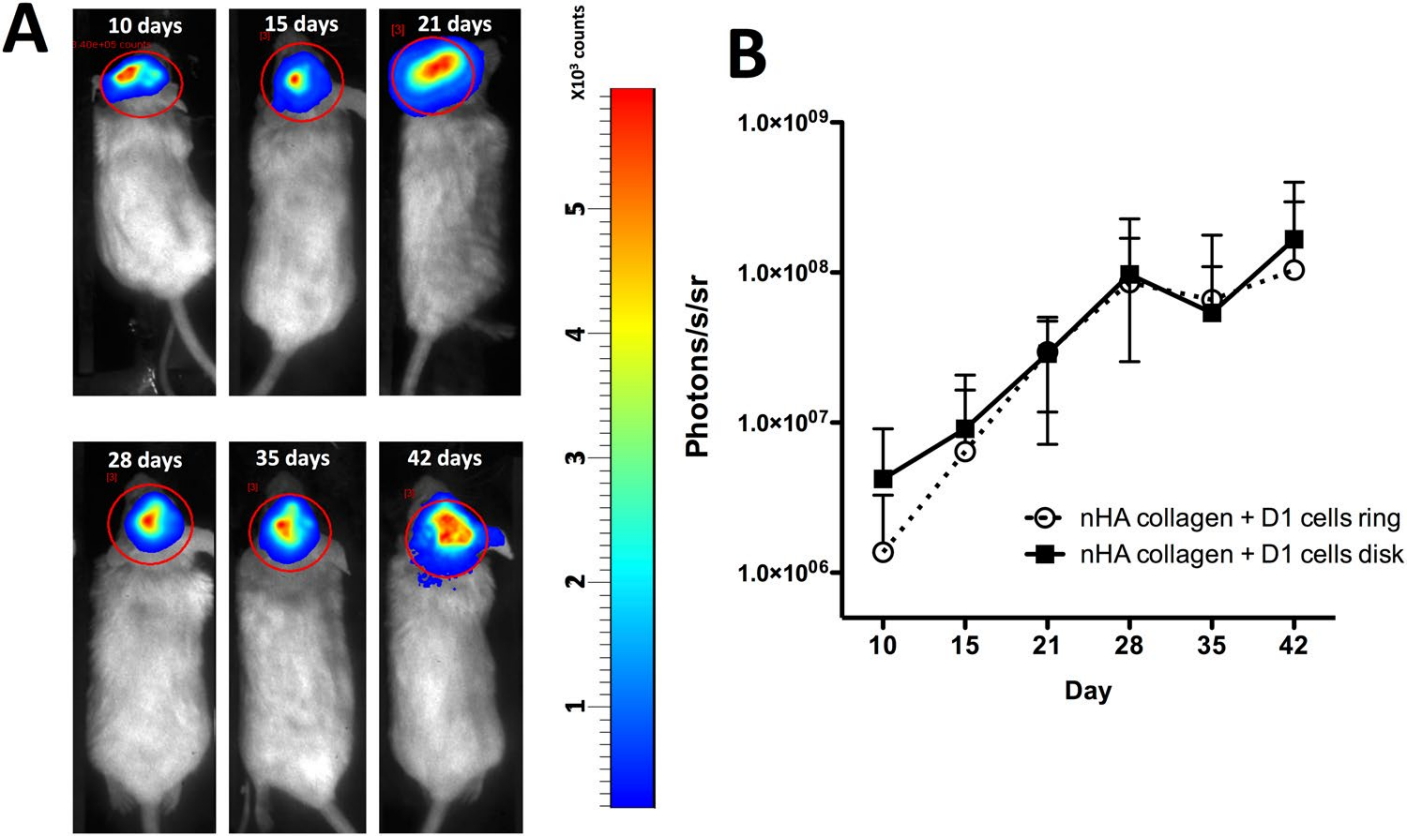
(**A**) Representative luminescence imaging of luciferase positive D1 cells in a ring geometry at 10, 15, 21, 28, 35 and 42 days post printing, in a mice calvaria model. (**B**) Quantification of the luciferase signal of luciferase positive D1 cells in a ring and disk geometry in a mice calvaria model (Average ± SD, n = 5).

In order to assess the proliferation of the printed cells, using the described printing design, we followed the luciferase signal of luciferase positive D1 cells in the *in vivo* calvaria model in mice up to 42 days. As observed in Fig. 4, a consistent signal augmentation can be observed towards time and no significant differences can be observed for both geometries tested.

## Bone repair following LAB

**Figure 5:**
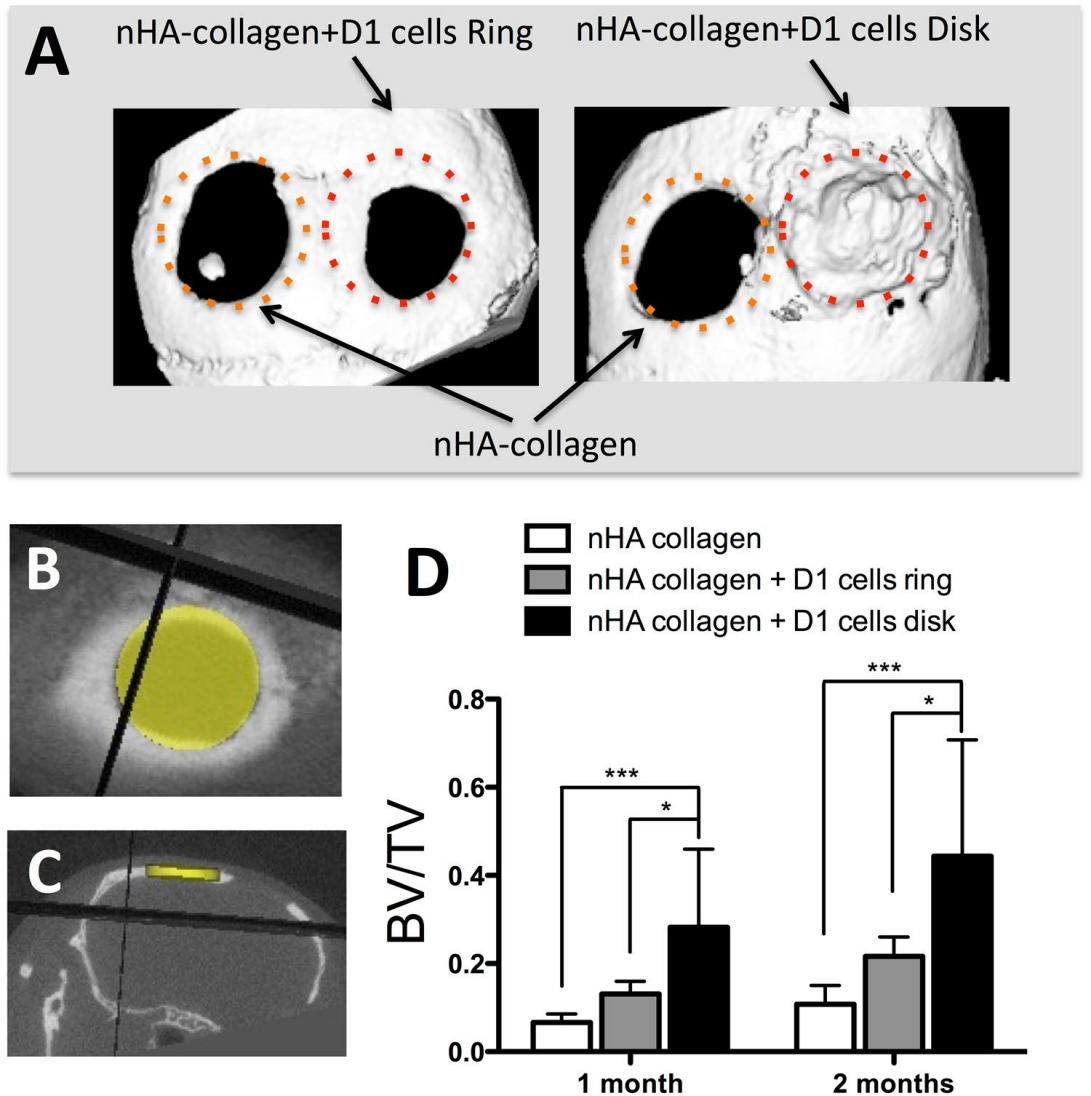
(**A**) Representative X-ray micro tomography (μCT) reconstruction images of nHA collagen and D1 cells printed in a ring or disk geometry (calvaria defect in the right side), or nHA collagen alone (calvaria defect in the left side), at 2 months post printing in a mice calvaria model. Horizontal (**B**) and coronal (**C**) μCT projection and regions of interest (3.3 mm diameter and 0.5 mm thick disk) for the evaluation of bone repair, in a calvaria defect in mice at 2 months post impression. (**D**) Quantitative assessment of bone volume/total volume (BV/TV) by μCT evaluation of nHA collagen and D1 cells, printed in a ring or disk geometry, or nHA collagen alone at 1 and 2 months post printing (Average ± SD, n = 9, *and ***denote p < 0.05 and p < 0.001, respectively).

The potential of different cell printed geometries was evaluated by assessing the bone repair, by X-ray micro tomography (μCT), at both 1 and 2 months post printing. As observed in Fig. 5, a marginal reconstruction of the defect, solely printed with nHA collagen ink can be observed at 2 months post printing (Fig. 5A and D). In the case of the defects printed with the nHA collagen material and with D1 cells in a ring geometry, again no major bone formation can be observed at both time points tested (Fig. 5A and D). Conversely, in the case of nHA collagen material and with D1 cells in a disk geometry, a significant increase in terms of bone formation can be observed at both 1 and 2 months, post printing, in relation to the two other tested conditions (Fig. 5A and D **)**.

**Figure 6:**
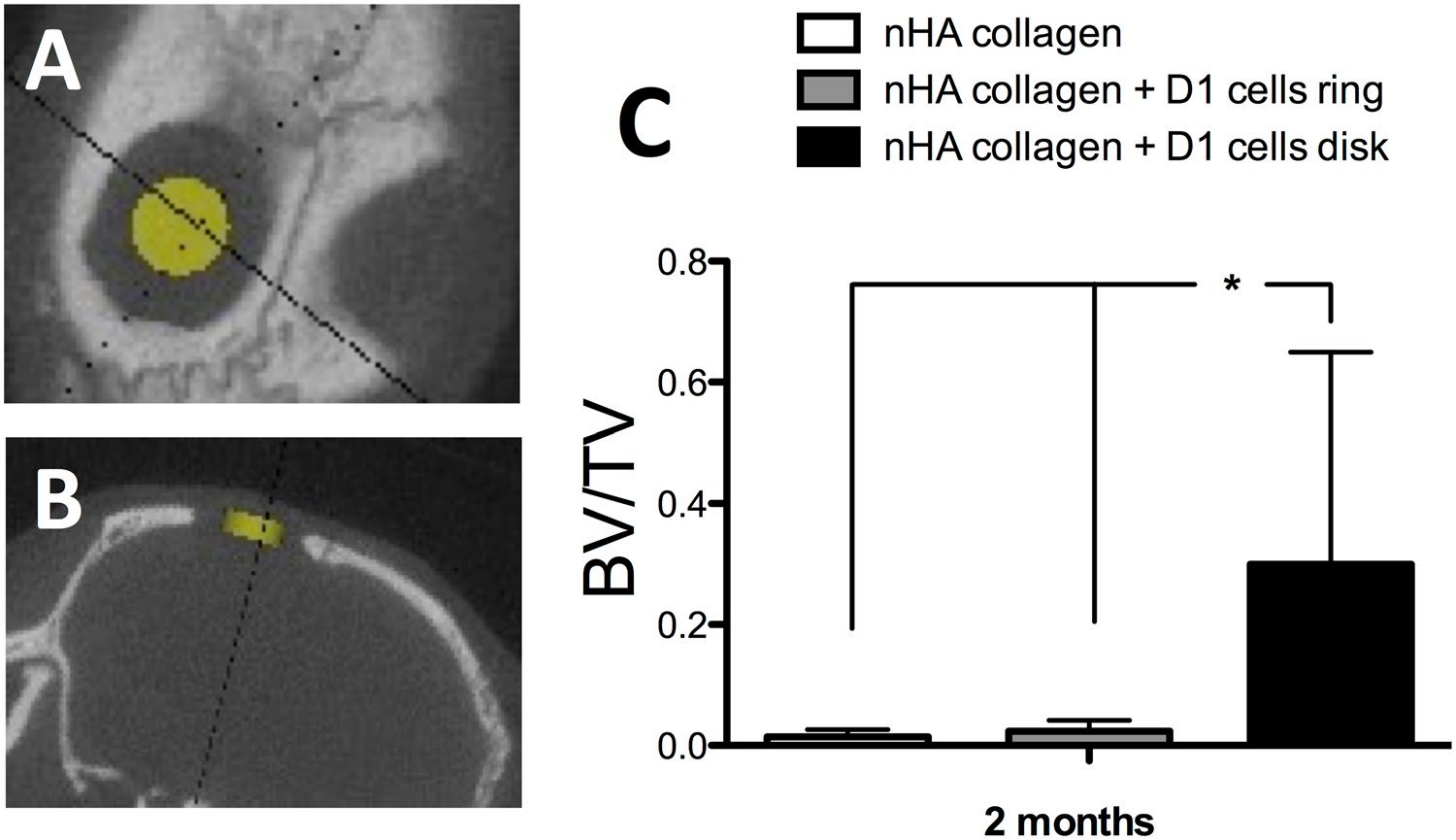
Horizontal (**A**) and coronal (**B**) X-ray micro tomography (μCT) projection and region of interest (1.5 mm diameter and 0.5 mm thick disk) for the evaluation of bone repair, in the center of a calvaria defect in mice at 2 months post impression. (**C**) Quantitative assessment of bone volume/total volume (BV/TV) by μCT evaluation of nHA collagen and D1 cells, printed in a ring or disk geometry, or nHA collagen alone at 2 months post printing (Average ± SD, n = 9, *denotes p < 0.05).

As means to further evaluate the impact of both cell printed geometries on bone repair we assessed bone repair, by μCT, at 2 months post printing using a 1.5 mm central region of the defect. As observed in Fig. 6C, a significant increase in terms of BV/TV can be observed in the case of the printing of nHA collagen and with D1 cells, in disk geometry, in comparison with the two other conditions. These results show that in the case of the disk printed geometry the regeneration is homogeneous throughout the defect, in contrast with the ring geometry, where regeneration is mainly observed at the periphery.

**Figure 7:**
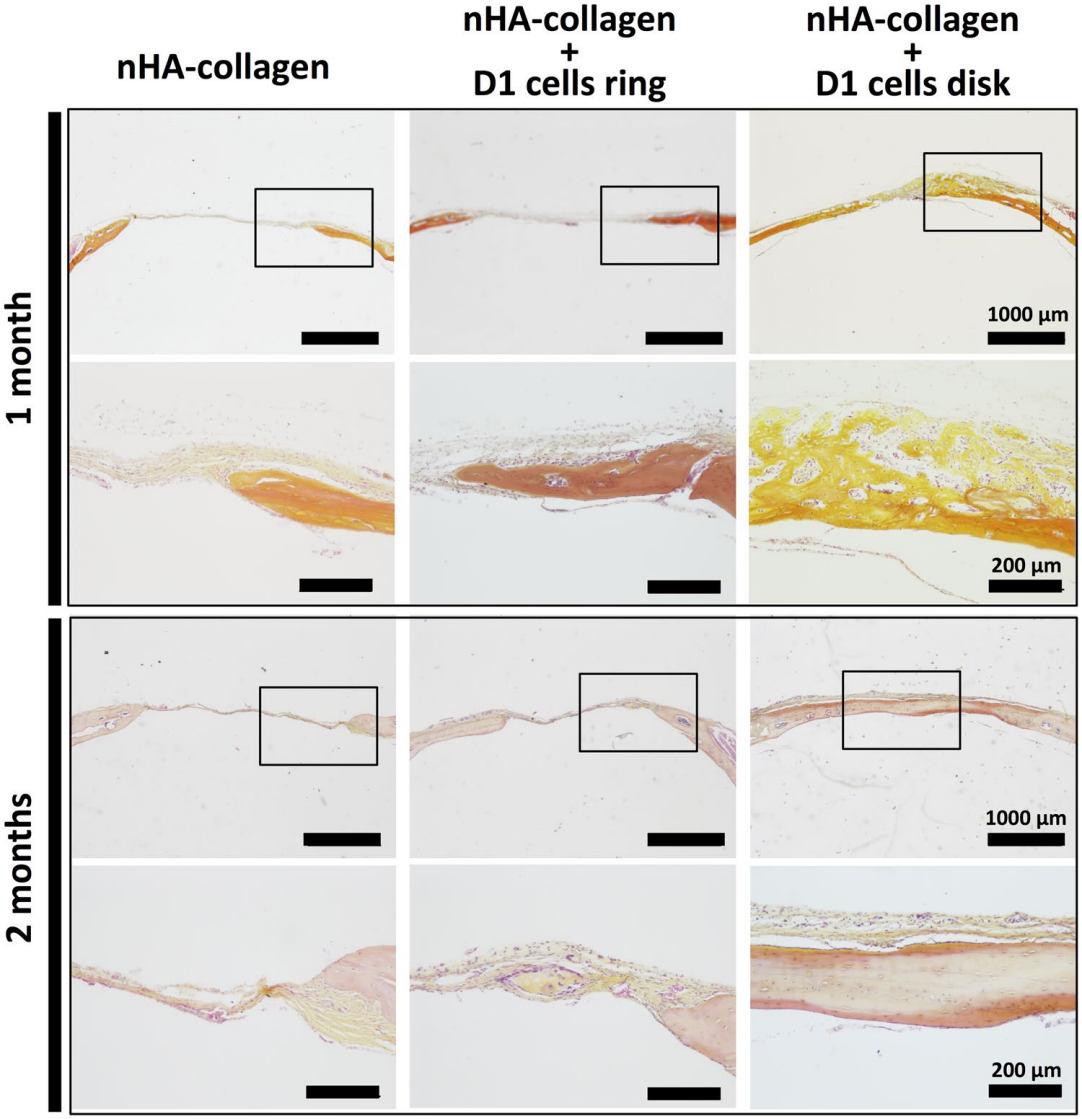
Histologic evaluation by Hematoxylin/Eosin/Safran (HES) staining of bone repair, in a calvaria defect in mice, at 1 and 2 months post impression of nHA collagen and D1 cells, printed in a ring or disk geometry, or nHA collagen alone. (Representative images are shown).

Micro-CT evaluation was confirmed by histological analysis of the samples recovered at both 1 and 2 months post printing, using hematoxylin eosin saffron (HES) staining. As observed in Fig. 7, at 1 month post printing, both nHA-collagen and nHA-collagen+ D1 cells, printed in a ring geometry, show only a marginal tissue reconstruction, particularly from the periphery of the defect. In contrast the nHA-collagen+ D1 cells, printed in disk geometry, show a substantial new bone formation, well distributed throughout the defect.

At two months post printing the same tendency was observed (Fig. 7), nHA-collagen printed condition showed little bone repair and the nHA-collagen+ D1 cells, printed in a ring geometry, condition showed to repair bone to some extent, but solely at the periphery of the defect. In contrast, nHA-collagen+ D1 cells, printed in disk geometry, show to the formation of mature bone, even in the center of the defect.

## Discussion

Current tissue engineering applications still lack the ability to organize cells within a 3D scaffold and to reproduce the microstructure of native tissues. In this sense bioprinting techniques have emerged. Bioprinters based on jetting, extrusion and LIFT methods can deposit biomaterials, viable cells and macromolecules as to generate complex 3D structures, which can mimic, to some extent, the architecture of native tissues^8^. We have previously demonstrated the feasibility of Laser-Assisted Bioprinting (LAB) for the printing of nano-hydroxyapatite (nHA), *in situ*, in a calvaria defect on mice^14^. We showed that, although with some variability, this approach could improve bone regeneration, while not inducing a deleterious effect on the adjacent brain tissue. Focusing on the refinement of this approach, here we combined the printing of nHA with mesenchymal stromal cells (MSCs, D1 cell line) and tested the impact of two different cell-printing geometries, with distinctive cellular repartitions (disc or ring), on bone repair.

MSCs are multipotent progenitor cells with the capacity to differentiate into a variety of cell types (*i.e*. osteoblasts, adipocytes, chondrocytes, tenocytes and skeletal myocytes), they have immunomodulatory properties, can be easily purified from different tissues (*e.g*. bone marrow, adipose tissue, umbilical cord) and have shown the capacity to secrete protective biological factors, making them as one of the most suitable cell source for tissue regeneration approaches^16, 17^.

As LAB is based on a nozzle-free hardware, it can avoid the problem of cell clogging, recurrent on other bioprinting approaches, while permitting to control the density and microscale distribution of cells, their viability and to attain higher speeds of deposition^18–21^. Also, due to its unprecedented printing precision, at the micron scale, LAB offers the opportunity to achieve an ultimate control over cell organization.

In this sense, Phamduy and colleagues showed that laser-based bioprinting could be used to create *ex vivo* models, based on spatially defined cancer cell printing over vascularized mesenteric tissue, relevant for the study of cancer cell dynamics during angiogenesis^22^.

Additionally, it was previously shown that human DermaMatrix scaffolds, printed with BMP-2 using inkjet technology, could favor bone repair upon implantation in a calvaria model in mice^23^. In this work the authors demonstrate the potential of inkjet printing in order to achieve the local arrangement of growth factors in order to attain guided bone repair. Nevertheless, this work was based on a complex two-step strategy, where the scaffold was initially printed *ex vivo* and then implanted. Also, the inherent risks of the use of growth factors in a wide range of clinical applications and due to the fact that they are only approved under very strict conditions^24^, may pose several constrains in order to translate to clinical applications. However, this work could show the interest of the patterning of bioactive molecules in order to achieve tissue regeneration.

Following this approach, here we focused on an *in situ* printing of MSCs, within a collagen and nHA matrix, in order to further improve the regeneration of a bone defect, while testing the impact of two different geometries of cell printing.

In line with previous reports^18–21^, LAB printed MSC cells show to remain viable and to proliferate, both *in vitro* and *in vivo*, independent of the geometry used. These results assert for the capacity of LAB to efficiently print mammalian cells with negligible effect on their viability and function. Indeed, we previously showed that by using the same LAB setting and the same cellular model, we could achieve a precise arrangement of MSC cells, without compromising cell viability, their capacity to proliferate and differentiate towards the osteoblastic lineage, and without inducing major DNA fragmentation^25^.

Ordered cellular patterns are found throughout nature, based on both the organization of the extracellular matrix and on the complex secretion of cellular morphogens and cell-to-cell interactions, able to generate bio/chemical gradients and mechanical signals that can lead to the establishment of organized tissue domains^26^. Focusing on the importance of this organization process, we tested two different morphologies, ring and disk, where the cell number and density were kept in the same range (from 700 to 800 cells/mm^2^, for ring and disk geometries, respectively). We could observe that a disk geometry was more favorable for *in vivo* bone regeneration, in the tested model. We hypothesize that this beneficial effect was based on an autocrine regulation by MSCs cells themselves. Indeed, several studies have shown that MSCs can regulate their differentiation, towards the osteoblastic lineage, via an autocrine/paracrine process by the secretion of interferon gamma and tumor necrosis factor alpha^27, 28^. We postulate that the disk cellular arrangement could sustain, due to the proximity and higher homogeneity of cell distribution, a paracrine regulation of MSCs differentiation towards the osteoblastic lineage. Based on *in vivo* evaluation we show that printed MSCs were capable to proliferate up to 42 days post implantation and to improve the regeneration of the calvaria defect, particularly for the cells printed in a disk geometry. However, here we did not evaluated the direct integration of the printed D1 cells on the regenerated bone tissue and future studies will shed new light on this subject. Nonetheless, this work provides the first report on the influence of cell printing geometry, *in situ*, for bone regeneration using LAB technology.

Previous reports on *in situ* bioprinting, using an inkjet technological approach, were focus on skin regeneration. Skardal and colleagues showed that amniotic fluid-derived stem cells *in situ* printing, over skin wounds, could improve the rate of wound closure^29^. This pivotal work shows the potential of bioprinting approaches to deliver cells in a fast, and off-the-shelf manner in order to be applicable in a clinical scenario. In the this work, we went a step further showing that in a more invasive chirurgical procedure, precise cellular deposition could be achieved and result in a significant improvement for tissue regeneration.

Technically, our approach allows to use, in the same platform, different ribbons with distinct cell types and bio inks. Ultimately this versatility can allow the applicability of bioprinting to other, more complex tissues, were additional components or cellular types can be organized in a 3D arrangement in order to favor tissue regeneration. Also, as this technique allows printing without direct contact we can envisage its application in a clinical scenario, in a sterile environment inside the operating room, where fully automatic robotic printers can be directed by the surgeon to achieve precise cellular implantation at the micron to millimeter scale. Nonetheless, the LAB technology is still restrained to flat surface applications and awaits new technological improvements in order to attain further complex tridimensional applications. Additionally, due to the versatility of LAB, the combination with bioactive factors and biomaterials can be achieved widening the perspectives for this bioprinting approach.

LAB presents several key factors that favor this approach in order to achieve defined, cellular based, tissue constructs. This is, to the best of our knowledge, the first report of *in situ* printing of mesenchymal stromal cells on a bone defect using LAB technology. This work opens new avenues on the development of bioprinting strategies for the building of tissues, from the ground up. Nonetheless, further technical developments are awaited in order to achieve large and more complex tissue reconstruction.

## Methods

### Reagents

Unless mentioned otherwise, all reagents were obtained from Sigma-Aldrich and were of analytical grade.

### Laser-assisted bioprinter (LAB)

The bioprinting laser-assisted workstation setup was in accordance on what previously described^15^. In brief, the laser source consisted on a solid Nd:YAG crystal laser (Navigator I, Newport Spectra Physics, 1064 nm, pulse duration of 30 ns, repetition rate of 1–100 kHz, mean power of 7 W). A scanning system, comprising two galvanometric mirrors (SCANgine 14, ScanLab), with a scanning speed attaining 2000 mm/s, was used to drive the laser beam. The laser beam focusing on the gold coating was achieved using a large field optical F-theta lens (58 mm focal length, S4LFT, Sill Optics, France). Additionally, a 5-placement carousel system was integrated into the workstation (NovaLase, S.A., Canéjan, France) enabling to print different bioinks and to form complex patterns. Dedicated software was used to control substrate positioning, carousel operation, pattern design and sample observation via video analysis. All experiments were performed at room temperature, in air, using a repetition rate of 1 kHz and with a distance of 1 mm between the ribbon and the receiving substrate. The laser energy deposit conditions were modulated by tuning the laser power (6–80 mW) or the diaphragm aperture (5–18 mm).

### Cell culture

The multipotent mouse bone marrow stromal precursor D1 cell line, obtained by ATCC, was used throughout this study. Cells were cultured on TCPS plastic with Dulbecco’s Modified Eagle Medium (DMEM, Gibco, Life Technologies), with 10% (v/v) fetal calf serum (FCS) (Lonza, Levallois Perret, France) at 37 °C and with 5% CO_2_. In order to allow the follow up of cells upon *in vitro* and *in vivo* printing the D1 cells were infected using lentiviral vectors containing the TdTomato protein gene (red) and the luciferase gene, under the control of the phosphoglycerate kinase promoter^30^ or the modified myeloid proliferative sarcoma virus promoter (MND)^31^, respectively. Briefly, for viral transduction, 2 × 10^5^ freshly trypsinized D1 cells were exposed to 6 × 10^6^ viral particles (multiplicity of infection (MOI) = 30). After 24 h in culture, virus-containing medium was replaced with fresh medium, and cells were expanded over several passages, using standard cell culture procedures.

### Bioink preparation

Cellularized bioink was composed of D1 cells at 120 × 10^6^ cells/mL in DMEM, supplemented with 10% (v/v) of FCS.

Collagen bioink was composed of a neutralized solution of type I rat collagen solution at 2 mg/mL (BD Biosciences, France). When indicated in the article, 1.2% (w/v) of nano hydroxyapatite (nHA) was equally dispersed inside the collagen matrix, and designated as nHA collagen. Nano hydroxyapatite was produced as previously described, *via* wet chemical precipitation^14^.

### Ribbon preparation

A thick gold film (50 nm) was deposited on a 3 cm diameter round quartz slide using a sputter coater (Emscope SC500, Quorum technologies, UK). Three microliters of bioink/cm^2^ were deposited on the metal film using a blade coater device (3570 Elcometer).

### Biopaper preparation

For *in vitro* printing, a 200 μm receiving layer (biopaper) was produced by spreading a neutralized and cold solution of type I rat collagen solution at 2 mg/mL (BD Biosciences, France) over a glass slide. Prior use, the glass slide was then placed in a 37 °C incubator during 2 hr in order to allow collagen to gel.

### Printing procedure

Two geometries of cellularized bioink printing were chosen in this work: a disk with 2 mm diameter and a ring with external and internal diameter of 3 and 2.1 mm, respectively. In order to maintain the same amount of cells both geometries were based on 50 impacts spots, were each impact contained around 50 cells (data not shown), and therefore each finished printed geometry corresponds to approximately 2500 cells. The final cell density corresponded to 693 or 796 cells/mm^2^, for ring or disk geometry, respectively. Based on previous reports^12, 18, 32^, the printing settings used were a speed of 300 μm/s, 1 kHz frequency, laser energy of 27.5 μJ and a gap distance (between the ribbon and the receiver layer, both *in vitro* and *in vivo*) of 1000 μm.

In the case of the nHA collagen bioink, each layer consisted on a disk design of 2 mm, with 50 impact spots, that was repeated 3 times, in order to attain a disk of approximately 100 μm. The printing settings used were a speed of 250 μm/s, 1 kHz frequency, laser energy of 50 μJ and a gap distance (between the ribbon and the receiver layer) of 1000 μm. For both geometries tested *in vivo* one layer was printed directly to the dura mater of the mouse and a second layer was printed over the cellularized printed ink geometry. In the case of the negative control, with no cells, only the two layers of nHA collagen were printed inside the defect.

### Metabolic activity assessment

Metabolic activity of D1 cells upon bioprinting, in a ring and disk geometry, was evaluated at 1 and 8 days, using a resazurin based assay^33^. Briefly, a solution of resazurin (0.1 mg/ml in PBS) was added to each well to a final 10% (v/v) concentration. After a 3 h incubation at 37 °C, 200 μl of the medium was transferred into a 96-well plate and fluorescence was measured (exc = 530 nm, em = 590 nm, Victor X3, Perkin Elmer). Results were expressed as percentage of metabolic activity of cells relative to day 1 in a ring geometry.

### Animal procedures

The procedures and mice handling were based on the principles of Laboratory Animal Care formulated by the National Society for Medical Research and approved by the Animal Care and Experiment Committee of University of Bordeaux, Bordeaux, France. Experiments were carried out in accredited animal facilities following European recommendations for laboratory animal care (EU Directive 2010/63/EU for animal experiments).

Sixty-four 12-week-old Balb/c female mice, weighting 19–20 g (Charles Rivers, France), were used in this study, ten for the *in vivo* assessment of D1 cell proliferation, *via* luciferase imaging, and 54 for the bone regeneration evaluation by micro tomography and histology. The different study groups were formed randomly.

For the establishment of the calvaria defect, the animals were first anesthetized with Ketamin (Imalgen, Merial, France) and Xylazine (Rompun, Bayer, France), through intraperitoneal injection, and after skin antisepsis (Betadine), an incision was performed in skull midline and the scalp was dissected to expose the calvaria. Then the periosteum membrane was carefully peeled off and two lateral 3.3 mm diameter circular bone defects were achieved using a 3.3 mm diameter trephine (Praxis l’instrumentiste, France). One defect was used for laser processing while the contralateral site was used as control. The surgical procedures were performed under constant saline irrigation and care was taken to prevent dura mater injury. Then, the animals were placed inside the bioprinting workstation for *in vivo* printing experiments. At the end of the experiment, the soft tissues were repositioned and sutured using 3/0 vicryl (Johnson and Johnson, USA). Animals recovered in a warm environment before being returned to animal facilities. At defined time points animals were sacrificed by CO_2_ inhalation and calvaria tissue was recovered and processed for X-ray microtomography and histologic analysis.

### *In vivo* bioluminescence imaging

*In vivo* bioluminescence imaging was conducted on a cryogenically cooled imaging system (PhotonIMAGER, Biospacelab, France) using the Photo-acquisition and 3D Vision software (Biospacelab, France). Briefly, animals implanted with luciferase positive D1 cells, were maintained under anaesthesia by isofurane inhalation and received an i.p. injection of an aqueous solution of the substrate D-luciferin (125 mg/kg, Promega). Then, they were placed in the imaging chamber and signal intensity was quantified as the sum of all detected photon counts, acquired during 30 seconds, within the region of interest after subtraction of background luminescence. The quantification of luminescence was performed at 10, 15, 21, 28, 35 and 42 days post printing.

### X-ray microtomography and analysis

Micro-CT was performed on Explore Locus SP X-ray μCT devices (General Electric, Milwaukee, WI) *ex vivo* with a source voltage of 80 kV and a current of 60 μA to obtain a 15 μm resolution from 900 X-ray radiographs with an exposure time of 3000 ms. After scanning, cross-sectional slices were reconstructed and three-dimensional analysis were performed using eXplore MicroView® software (General Electric Healthcare, Milwaukee, WI). Reconstruction of the region of interest was performed after correction of the center of rotation and calibration of mineral density. Each scan was reconstructed using the same calibration system to distinguish bone and air. Mineral Content (MC) and Mineral Density (MD) volume were measured for each group. After scanning, cross-sectional slices were reconstructed and three-dimensional analyses were performed using Microview® software.

### Histological analysis

The animal’s skull were harvested, fixed with 4% (w/v) paraformaldehyde for 24 hr at 4 °C and then demineralized during 1 h (DC3 QPATH, VWR, France), dehydrated and embedded in paraffin. Ten microns coronal sections were cut and stained with hematoxylin–eosin–saffron (HES), using standard protocols, and observed under a photomicroscope (Nikon eclipse 80i, The Netherlands).

### Statistical analysis

Sample sizes were chosen to ensure adequate power (>85%, at significance of 0.05) to detect predicted effect sizes, which were estimated on the basis of either preliminary data or previous experiences with similar experiments.

Using the Graphpad Prism 5.0 software, a D’Agostino and Pearson omnibus normality test was used in order to test if data obeyed to a Gaussian distribution. Statistically significant differences between several groups were analyzed by the non-parametric Kruskal-Wallis test, followed by a Dunns post-test. The non-parametric Mann-Whitney test (two-tailed) was used to compare two groups. A p value lower than 0.05 was considered to be statistically significant.
